# Multifaced roles of adipokines in endothelial cell function

**DOI:** 10.3389/fendo.2024.1490143

**Published:** 2024-11-04

**Authors:** Yu Yan, Lihui Wang, Ni Zhong, Donghua Wen, Longhua Liu

**Affiliations:** ^1^ School of Exercise and Health, Shanghai University of Sport, Shanghai, China; ^2^ Department of Radiology, Shanghai East Hospital, Tongji University, Shanghai, China; ^3^ Department of Laboratory Medicine, Shanghai East Hospital, Tongji University School of Medicine, Shanghai, China

**Keywords:** endothelial dysfunction, atherosclerosis, adipokines, cardiovascular diseases, obesity

## Abstract

Obesity significantly contributes to the progression of cardiovascular diseases (CVDs) and elevates the risk of cardiovascular mortality. Atherosclerosis, the primary pathogenic process underlying CVDs, initiates with vascular endothelial dysfunction, serving as the cornerstone of vascular lesions. Adipokines, bioactive molecules secreted by adipose tissue that regulate metabolic and endocrine functions, play a pivotal role in modulating endothelial function during atherosclerosis. This review comprehensively examines the distinct roles of various adipokines in regulating endothelial function in atherosclerosis. We categorize these adipokines into two main groups: protective adipokines, including adiponectin, FGF21, CTRP9, PGRN, Omentin, and Vaspin, and detrimental adipokines such as leptin, Chemerin, Resistin, FABP4, among others. Targeting specific adipokines holds promise for novel clinical interventions in the management of atherosclerosis-related CVDs, thereby providing a theoretical foundation for cardiovascular disease treatment strategies.

## 1 Introduction

Cardiovascular diseases (CVDs) continue to be the primary cause of death for people, and the total number of people suffering from these diseases is steadily increasing as the population expands and ages (1). Atherosclerosis initially begins with endothelial dysfunction, secretion of chemokines and growth factors as a result of endothelial cell dysfunction induces recruitment of monocytes to the subendothelial region, smooth muscle cell proliferation, and increased matrix protein synthesis. Subsequently, recruited monocytes differentiate to macrophages, accumulating lipids, and eventually progressing into foam cells. As atherosclerosis advances, it compromises arterial lumen diameter, diminishes blood flow within arteries, and culminates in occlusion of affected arteries (2).

Endothelial dysfunction stands out as a key feature in the transition from early atherosclerosis to eventual vascular occlusive infarction, marking it as a fundamental aspect of atherosclerosis (3). Atherogenic elements such as oxidized low-density lipoprotein (oxLDL),free fatty acids and homocysteine contribute to oxidative stress in endothelial cells (4). Increased reactive oxygen species (ROS) generated by oxidative stress will cause the oxidation of nucleic acids, lipids, and proteins, disrupting vascular homeostasis (5). Inflammation within the vasculature is crucial to both the onset and advancement of atherosclerosis. Damaged endothelium becomes activated, secreting inflammatory factors such as monocyte chemotactic protein-1 (MCP-1), intercellular adhesion molecule-1 (ICAM-1), vascular adhesion molecule-1 (VCAM-1), and others. These substances draw monocytes, which adhere to the endothelial cells that have been activated. The leukocytes then roll and move into the subendothelial region, piercing the artery wall and causing inflammation (6). Endothelial injury prompts apoptosis, and numerous atherogenic factors —such as elevated levels of LDL, oxLDL, and oxidative stress—can induce the programmed death of endothelial cells. In advanced stages, extracellular vesicles derived from endothelial cells during apoptosis drive the progression of atherosclerosis (7). Within endothelial cells, eNOS catalyzes nitric oxide (NO) production. However, eNOS uncoupling can be another major cause of endothelial dysfunction, which diminishes NO production and intensifies oxidative stress. eNOS uncoupling occurs when eNOS transitions from producing NO to generating superoxide anions (O2–) in the presence of molecular oxygen, a process commonly associated with cardiovascular conditions like atherosclerosis and diabetes (8). Moreover, in advanced stages of atherosclerosis, increased platelet-endothelial interactions can exacerbate endothelial dysfunction, leading to an overproduction of prothrombotic molecules, including thrombin and PAI-1, which cause plaques to become unstable and prone to rupture (9).

Obesity is a chronic metabolic disorder caused by an abnormal accumulation of adipose tissue, increasing the risk of developing cardiometabolic conditions. A persistent low-grade inflammatory state in adipose tissue caused by obesity is closely associated with the onset of cardiovascular disease (10). Adipocyte hypertrophy and hyperplasia, increased inflammation, irregular extracellular matrix remodeling, fibrosis, and modified adipokine production are some of the factors behind adipose tissue dysfunction (11).

Adipokines are peptides that convey information about the functional state of adipose tissue to various target such as Adiponectin, leptin, fibroblast growth factor 21, retinol-binding protein 4 and vaspin (12), they are crucial in maintaining energy and vascular homeostasis, exerting their effects on both nearby and distant tissues through autocrine and endocrine pathways. Adipokines have been shown in prior research to play a critical role in the development and reversal of atherosclerosis, and some are considered direct mediators connecting obesity to atherosclerosis, as they impact endothelial cell function within the vessel walls (13, 14). Adipokines differ in their involvement in endothelial function and the development of atherosclerosis. In this review, we will comprehensively review the role of adipokines in regulating endothelial function and atherosclerosis.

## 2 Protective adipokines

### 2.1 Adiponectin

Adiponectin is a 30-kDa multimeric protein primarily released by white adipose tissue. By interacting with its receptors, T-cadherin, AdipoR1, and AdipoR2, it controls the metabolism of fat and glucose (15, 16). Adiponectin has anti-atherogenic properties which can slow the advancement of atherosclerosis. In individuals with cardiovascular disease or dyslipidemia, adiponectin levels had a negative correlation with hs-CRP and a positive correlation with HDL-C, and sVCAM-1 (17).

**Figure 1:**
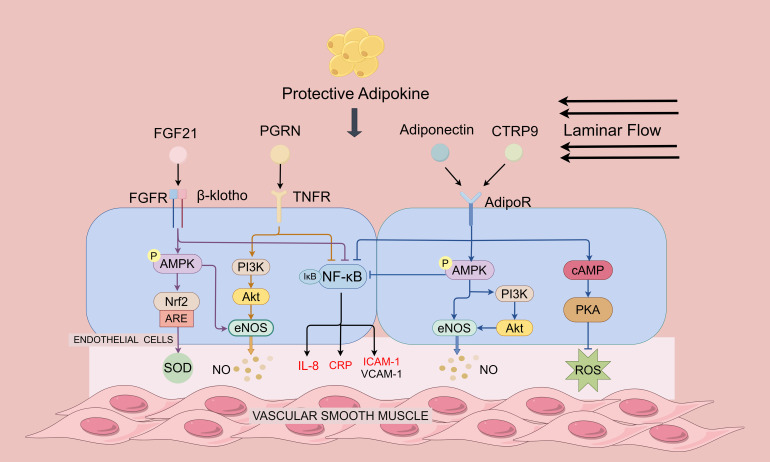
Protective adipokines in regulating endothelial function and atherosclerosis. Protective adipokines depicted are adiponectin, CTRP9, FGF-21 and PGRN. Adiponectin and CTRP9 can activate the AMPK pathway by binding to the receptor AdipoR1: On the one hand, AMPK directly activates eNOS to promote NO production; on the other hand, AMPK activates Akt and phosphorylates eNOS through the promotion of P13 kinase, and the activation of AMPK also leads to a decrease in NF-κB activity. Furthermore, it inhibits ROS generation in endothelial cells through cAMP/PKA-dependent signaling. PGRN directly binds to TNF receptor (TNFR), inhibits NF-κB activation, and ameliorates endothelial inflammation. PGRN also activates PI3 kinase and upregulates NO levels through the Akt/eNOS pathway. FGF21 enhances AMPK activity by binding to a complex of receptors FGFR and β-klotho. On the one hand, AMPK upregulates Nrf-2 expression to eliminate oxidative stress. On the other hand, AMPK directly enhances eNOS activity thereby increasing NO production and endothelium-dependent restoration of vasodilation. FGF21, PGRN, Adiponectin and CTRP9 inhibit NF-κB activation and reduces CRP, IL-8 and adhesion molecules expression by binding to the receptor.

Adiponectin significantly ameliorates the oxLDL-induced reduction in eNOS activity in atherosclerosis via the AMPK-PI3K-AKT-eNOS signaling pathways (**Figure 1**). Overexpression of adiponectin in ApoE^-/-^ mice significantly reduced atherosclerotic plaque area and upregulated eNOS expression in the aorta (18). Hattori et al. found that HMW adiponectin activates AMPK, thereby enhancing eNOS phosphorylation and NO production in endothelial cells (19). AMPK promotes Akt activation, eNOS phosphorylation, and angiogenesis through the promotion of PI3K. Furthermore, in isolated aortic rings from caveolin-1 mutant mice, adiponectin phosphorylates eNOS in adipoR1/caveolin-1-dependent manner to increase endothelium-dependent vasorelaxation and endothelial NO generation (20).

Adiponectin prevents monocyte adherence to endothelial cells and adhesion molecule production that is stimulated by TNF-α. Compared to WT mice, Adiponectin-KO animals exhibited considerably higher levels of VCAM-1 and ICAM-1. This increase may be attributed to adiponectin’s suppression of TNF-α-induced NF-κB activation (21). Noriyuki Ouchi et al. and Devaraj S et al. demonstrated that adiponectin treatment significantly inhibited NF-κB activity in HAEC, whereas AMPK activation decreased NF-κB activity and prevented CRP generation and secretion induced by high glucose (22, 23). NF-κB also stimulates the expression of IL-8, an important mediator of monocyte chemotaxis and firm adhesion to endothelial cells, an essential process in the initial stages of atherosclerosis. IL-8-mediated adiponectin exerts anti-inflammatory actions through PKA-regulated NF-κB inhibition and activation of PI3K/Akt (24, 25). When oxLDL binds to LOX-1, it activates MAPK (mitogen-activated protein kinase), which consequently increases the MCP-1 expression. However, Mutual regulation between lipocalin and LOX-1 exacerbates oxidative stress and ox-LDL uptake, leading to endothelial dysfunction in atherosclerosis. In ApoE KO mice, adiponectin reverses elevated LOX-1 expression and reduces NF-κB expression in the aorta (26). In cultured endothelial cells, adiponectin activates the PI3K/Akt signaling pathway through the receptor CRT’s junction protein CD91, induces COX-2 expression, activates the PGI2 pathway, and inhibits platelet and leukocyte adhesion to activated endothelial cells (27). Adiponectin enhances COX-2 expression via the SphK-1-S1P signaling pathway. Ikeda et al. found that in rat neonatal cardiomyocytes, inhibition of SphK-1 treatment decreased adiponectin-induced COX-2 expression (28).

Adiponectin induces endothelial cell migration, angiogenesis, and human umbilical vein endothelial cells (HUVEC) differentiation into capillary-like formations. In cultured endothelial cells, adiponectin prevented injury-induced intimal thickening by inhibiting HB-EGF expression in endothelial cells of injured vessel walls (29). Endothelial cell pyroptosis induces a cellular inflammatory response that promotes the atherosclerotic process. Adiponectin treatment reduced FoxO4 expression in HAEC exposed to LPS and inhibited the activation of NLRP3 inflammatory vesicles, which in turn inhibited the pyroptosis of aortic endothelial cells (30). In cultured HUVECs, recombinant adiponectin inhibits apoptosis and increases cell viability (31). In addition, adiponectin’s protective effect against Ang II-induced endothelial injury relies on its interaction with the cell-surface receptor adipoR1. Adiponectin regulates apoptotic pathways by decreasing LOX-1 expression and upregulating the ratios of cIAP-1 and Bcl-2/Bax (32).

The protein hydrolysis cleavage product of Adiponectin, gAD, has a higher binding affinity for adipoR1 and inhibits hyperglycemia-induced apoptosis in HUVEC cells by partially linking to adipoR1. In cultured HUVEC, gAP exerts a partial anti-apoptotic effect by stimulating the phosphorylation of AMPK at the Thr176 site, increasing eNOS activity, enhancing NO production, and inhibiting ROS production (33, 34) (**Table 1**, adiponectin).

**Table 1:** Protective adipokines in regulating endothelial function and atherosclerosis.

Adipokines	Major function	Mechanism	Reference
Adiponectin	↓monocyte adhesion ↑angiogenesis and endothelial cell migration ↓apoptosis ↓oxidative stress ↓inflammation ↓pyroptosis ↑eNOS activity and NO production	Adiponectin- AMPK-NO Adiponectin-AMPK-P13K-Akt-eNOS Adiponectin-Sphk1-S1P-Cox-2 Adiponectin-CRT/CD91-Cox-2-PGI2 Adiponectin- LOX-1	(15, 17, 20–22, 24, 25, 27, 30–32, 34, 35)
FGF21	↓inflammation ↓oxidative stress ↓monocyte adhesion ↓apoptosis ↓pyroptosis ↑eNOS activity and NO production ↓senescence ↑sensitivity to Ach-induced vasorelaxation	FGF21-PI3K-Akt-Fox3a FGF21-β-klotho-FGFR-CaMKK-AMPK FGF21-TET2-UQCRC1-ROS FGF21-FGFR1-Syk-NLRP3-ASC FGF21-Fas/FADD FGF21-NF-κB FGF21-Sirt1	(36–44)
CTRP9	↓apoptosis ↑angiogenesis and endothelial cell migration ↓inflammation ↓senescence ↓oxidative stress ↑eNOS activity and NO production	CTRP9-AMPK-HDAC7-p38 MAPK CTRP9-SIRT1-PGC1α-AMPK-ACC-NF-κB CTRP9-PGC-1α-AMPK-eNOS CTRP9-AMPK-KLF4	(45–49)
PGRN	↓monocyte adhesion ↑eNOS activity and NO production ↓inflammation ↑vasorelaxation	PGRN-TNFR-NF-κB PGRN-EphA2-Akt/NF-κB PGRN-Akt-eNOS PGRN-EphA2/Sotrilin1-eNOS	(50–53)
Omentin	↑monocyte adhesion ↓oxidative stress ↓vasodilation ↓inflammation ↓thrombosis ↑apoptosis	Omentin-ERK-NF-κB Omentin-AMPK-PPARδ-ROS Omentin-AMPK-PPARδ-Akt/eNOS-NO Omentin-AMPK-JNK	(54–60)
Vaspin	↑eNOS ↓apoptosis ↓monocyte adhesion ↓inflammation ↑vasodilation	Vaspin-STAT3 Vaspin-PI3K-Akt Vaspin-AMPK-NF-κB	(61–64)

↑, increase; ↓, decrease.

### 2.2 FGF21

Fibroblast growth factor 21 (FGF21) was first found to be secreted by brown adipose tissue (BAT) during thermogenic activation; in addition, it is also primarily secreted by the liver (65). FGF21 acts as an independent protective factor to reduce atherosclerotic injury and prevent endothelial dysfunction. FGF21 deficiency can lead to systemic inflammation that promotes the progression of atherosclerosis (36).

FGF21 can inhibit vascular endothelial inflammation by suppressing NF-κB pathway. Compared with normal rats, atherosclerotic rats had lower expression levels of FGF-21 in vascular endothelial cells. Additionally, *in vitro* injection of FGF-21 led to a reduction in lipid levels, Rho-kinase activity, and NF-κB expression in rats (37). The expression of inflammatory markers was increased in the aorta of apoE/FGF21 DKO mice with increased circulating concentrations of ICAM-1, VCAM-1, and TNF-α compared with apoE KO mice (36, 66).

FGF21 attenuates oxidative stress in the vascular endothelium and inhibits endothelial cell apoptosis. Ouyang et al. found that FGF21 treatment attenuates uric acid (UA)-induced endoplasmic reticulum stress, and inflammation through activation of SIRT1 (38). FGF21 acts by interacting with a complex receptor that includes FGFR and β-klotho (66). FGF21 binds to FGFR to enhance CaMKK2 and AMPKα activity (39). AMPKα promotes the expression of CAT, Nrf-2, and HO-1 to eliminate oxidative stress. Additionally, AMPKα directly enhanced eNOS activity to increase NO production and endothelium-dependent restoration of vasodilation. In a rat model of atherosclerosis, FGF-21 increased SOD levels, glutathione levels, and decreased malondialdehyde levels. This was mediated by inhibiting caspase3, suppressing p38 activation, and decreasing apoptotic activity, as evidenced by the increased ratio of BCL-2 to BAX expression (40).

In atherosclerosis, ox-LDL upregulates the expression of Fas apoptotic protein and promotes the interaction between Fas to its ligand FasL, which mediates apoptosis. In ox-LDL-incubated CMEC, increased levels of FGF21 reduced CMEC apoptosis (41). FGF21 inhibited atherosclerosis through mitigating Fas-mediated apoptosis in ApoE^-/-^ mice, and *in vitro* studies have found that FGF21 inhibited apoptosis and prevented atherosclerosis progression in HUVEC through the Fas/FADD mechanism (42). By inhibiting the activation of the MAPK signaling pathway, FGF21 increased the cell survival of HUVECs and provided protection against H_2_O_2_-induced apoptosis (40).

FGF21 binding to FGFR1 inhibits phosphorylation of SYK Tyr525/526, which inhibits activation of the NLRP3 inflammasome, reduces vascular endothelial cell pyroptosis, thereby exerting anti-atherosclerotic effects (43). In addition, FGF21 inhibits HUVEC cell death through the TET2-UQCRC1-ROS pathway. FGF21 upregulates UQCRC1, which is downregulated by ox-LDL, and reduces UQCRC1 silencing-induced HUVEC cell death and ROS production (44). FGF21 also exerts its antiatherogenic effects through an adiponectin-dependent mechanism (66) (**Table 1**, FGF21).

### 2.3 CTRP9

C1q tumor necrosis factor-related protein 9(CTRP9), a recently identified adipokine, has the potential to inhibit atherosclerotic plaque formation. Clinical research has indicated that myocardial infarction patients have lower serum levels of CTRP9. Meanwhile, exogenous CTRP9 inhibited atherosclerotic plaque formation in ApoE KO and CTRP9 KO mice (45). CTRP9 binds to the receptors AdipoR1 and N-cadherin to activate multiple signaling pathways and regulate endothelial function.

CTRP9 inhibits vascular endothelial inflammation and leukocyte endothelial adhesion. CTRP9 treatment significantly diminished TNFα-induced NF-κB activation and lowered the expression levels of ICAM-1 and VCAM-1 (46). Meanwhile, CTRP9 ameliorated endothelial inflammation-mediated attenuation of AMPK signaling and attenuated endothelial senescence by restoring autophagy and autophagic flux via AMPK activation (47).

CTRP9 can promote eNOS phosphorylation through the AMPK pathway. On the other hand, CTRP9 phosphorylates HDAC7 and p38 MAPK via AMPK, thereby influencing the pro-angiogenic transcription factor MEF2. It also enhances VEGF production and secretion to induce angiogenesis (45). In aortic rings isolated from wild-type mice, CTRP9 may exert vasoprotective effects through the AdipoR1/AMPK/eNOS/NO signaling pathway that is approximately 3-fold more effective than that of adiponectin (67). In HUVEC, CTRP9 stimulates eNOS activation via AMPK or Akt signaling pathways, thereby improving endothelial function and supporting hemotransfusion following ischemia. Sun et al. demonstrated that CTRP9 attenuated ox-LDL-induced endothelial damage and counteracted ox-LDL-mediated reduction in HUVEC proliferation, and angiogenesis through PGC-1α/AMPK-activated antioxidant enzyme induction (48). Overexpression of CTRP9 inhibits vascular senescence in ApoE-KO diabetic mice and reduces atherosclerotic plaque formation (49) (**Table 1**, CTRP9).

### 2.4 PGRN

Progranulin (PGRN) functions as an autocrine growth factor that participates in multiple pathophysiological processes such as atherosclerosis. PGRN ameliorates endothelial inflammation through the NF-κB pathway while inhibiting neutrophil chemotaxis. PGRN directly binds to the TNF receptor (TNFR), inhibiting TNF-α-activated intracellular signaling, thereby inhibiting NF-κB activation, blocking monocyte attachment to HUVEC, decreasing the expression of VCAM-1, ICAM-1, TNF-α and MCP-1 that ameliorated inflammation. In comparison to ApoE KO mice, PGRN/ApoE DKO mice had more severe atherosclerosis, enhanced levels of adhesion molecules and reduced expression of endothelial eNOS in aortic lesions (50). *In vitro*, PGRN treatment of hBMVEC reduced TNF-α-induced ICAM-1 expression (51). Tian et al. found that RhPGRN countered the reduction in cell viability and migration caused by high uric acid (Hcy) through the PGRN/EphA2/AKT/NF-κB pathway and increased endothelial barrier function by modulating VCAM-1 and VE-cadherin expression recovery (68).

PGRN upregulates nitric oxide (NO) levels in HUVECs by acting the Akt/eNOS pathway. PGRN activates via EphrinA2 and Sortilin1 receptors and endothelial eNOS to regulates vascular tone and blood pressure (52). On the other hand, PGRN pretreatment enhanced acetylcholine (ACh)-induced endothelium-dependent relaxation and cGMP production induced by the NO donor sodium nitroprusside (SNP). PGRN enhances ACh-induced NO-mediated relaxation by increasing cGMP production in smooth muscle (53) (**Table 1**, PGRN).

### 2.5 Omentin

Omentin is a newly discovered adipokine mainly expressed in visceral adipose tissue (VAT). Coronary endothelium has significantly lower levels of Omentin-1 compared to non-CAD patients (54).

Omentin attenuates leukocyte adhesion to the endothelium. Omentin decreased the expression of ICAM-1 and VCAM-1 in HUVEC, which may inhibit expression of adhesion molecules by inhibiting the ERK/NF-κB pathway (55). In addition, Omentin-1 inhibited p53 phosphorylation to restore ox-LDL-induced KLF2 levels, and thereby inhibited KLF2-mediated proinflammatory cytokine-induced expression of VCAM-1, significantly reducing leukocyte adhesion to HUVEC (56).

Omentin increases NO bioavailability and promotes vasorelaxation. Omentin-1 increases NO metabolites within the aorta and significantly increases the ratio of phosphorylated eNOS to total eNOS, increasing NO bioavailability, increasing NO-dependent vasorelaxation of the aorta and improving its endothelial function (57). On the other hand, Omentin inhibits COX-2 and exerts anti-inflammatory effects by activating the AMPK/eNOS/NO pathway and preventing JNK phosphorylation (58).

Omentin reduces endothelial oxidative stress. Kamaruddin et al. found that Omentin inhibited ROS production in HUVECs and significantly increased GPx activity, protecting HUVECs from H_2_O_2_-induced apoptosis (59). Liu et al. found that Omentin-1 prevents high glucose-induced vascular endothelial dysfunction by activating the AMPK/PPARδ pathway, inhibiting oxidative stress, and boosting NO production (60). *In vitro*, Omentin-1 effectively facilitates the formation of vascular-like structures and attenuates apoptosis, and Omentin stimulates the Akt/eNOS signaling pathway and activates AMPK to improve revascularization (69) (**Table 1**, Omentin).

### 2.6 Vaspin

Vaspin, a recently discovered adipokine classified within the serine protease inhibitor family, exhibits multifaceted effects on various physiological processes. Notably, it plays a pivotal role in delaying macrophage apoptosis, thereby impeding endoplasmic reticulum stress-induced apoptosis in apoE^-/-^ mouse models, consequently mitigating atherosclerotic plaque progression (70).

Vaspin improves endothelium-dependent vasodilation by activating the STAT3 signaling pathway. This activation leads to altered DDAH II expression, which lowers asymmetric dimethylarginine (ADMA) levels and promotes the phosphorylation of eNOS (71). Additionally, by inhibiting acetylcholinesterase (AChE) activity, vaspin increases endothelium-dependent relaxation mediated by NO. Jung et al. found that vaspin protects vascular endothelial cells against apoptosis by enhancing the activity of the PI3K/Akt signaling pathway (61).

Moreover, Vaspin has anti-inflammatory properties; in a concentration-dependent manner, it prevents TNF-α and IL-1-mediated activation of NF-κB and its downstream components, shielding endothelial cells from pro-inflammatory cytokine-induced inflammation (62). Vaspin may also reduce cytokine-induced adhesion molecules through inhibition of AMPK-activated NF-κB gene expression and reduce TNFα-induced monocyte adhesion to HAECs (63) (**Table 1**, Vaspin).

## 3 Deteriorative adipokines.

### 3.1 Leptin

The hormone leptin is generated by white adipose tissue and its receptors are expressed in many tissues including the cardiovascular system (72). Under hyperleptinemic conditions, leptin upregulates caveolin-1 protein expression, which activates the ERK1/2 and eNOS pathways and damages vascular endothelial cells (73). Leptin promotes endothelium imbalance in obese mice by upregulating eNOS expression and lowering intracellular L-arginine. This leads to eNOS uncoupling, endothelial NO depletion, and a rise in cytotoxic ONOO. Thus, an imbalance between eNOS expression and intracellular L-arginine levels may be one of the potential pathways of leptin-induced endothelial dysfunction (74).

Leptin induces pro-platelet and leukocyte adhesion to activated endothelial cells. Research conducted *in vitro* has revealed that leptin stimulates ERK1/2 to enhance the production of PAI-1, thereby facilitating the formation of plaque (75). Cirillo et al. demonstrated that leptin increases leukocyte adherence on the surface of human coronary artery endothelial cells (HCAECs) by promoting the production of tissue factor (TF) and cell adhesion molecules (CAMs) via the NF-κB pathway (76). In addition, leptin induces CRP expression. On the one hand, Leptin induces CRP expression in HCAEC by activating receptors to increase ROS production and ERK1/2 phosphorylation; on the other hand, Leptin may directly induce IL-6 production, which further upregulates CRP production in the liver. CRP activates the complement system, induces ICAM-1, VCAM-1, decreased eNOS, tPA activity, and platelet regulatory protein expression to produce proatherogenic effects on vascular endothelial cells (77).

Within endothelial cells, ROS serve as secondary messengers in the signaling pathways that are triggered by leptin. Yamagishi et al. found that leptin increased fatty acid oxidation through PKA in bovine aortic endothelial cells (BAEC), which in turn enhanced mitochondrial superoxide production and MCP-1 expression (78). In HUVEC, leptin increased ROS accumulation and activated the JNK pathway. Additionally, leptin activated NF-κB, which was activated in response to increased MCP-1 expression in HUVEC. Persistent oxidative stress in endothelial cells due to elevated leptin levels may initiate the development of atherosclerosis (79).

Leptin also has pro-endothelial angiogenic effects. By activating the Leptin receptor (Ob-R), leptin stimulates the angiogenic process and produces growth signals via tyrosine kinase-dependent intracellular pathways. Studies *in vitro* and *in vivo* have confirmed that leptin activates endothelial Ob-R and induces HUVEC proliferation and elevated expression of MMP-2 and MMP-9, which mediate aberrant angiogenesis (80). Garonna et al. found that leptin binding to the leptin receptor Ob-Rb stimulated VEGFR2 phosphorylation and enhanced COX-2 and PI3K/Akt expression in the endothelium through activation of p38MAPK, accelerating EC proliferation and capillary formation (81). It should be mentioned that another study found that leptin controls vascular tone via triggering the Akt-eNOS phosphorylation pathway, which in turn causes the production of NO (82) (**Table 2**, Leptin).

**Table 2:** Deteriorative adipokines in regulating endothelial function and atherosclerosis.

Adipokines	Major function	Mechanism	Reference
Leptin	↑imbalance between eNOS expression and intracellular L-arginine levels ↑oxidative stress ↑angiogenesis ↑monocyte adhesion ↑thrombosis ↑inflammation	Leptin-JAK2-STAT3-ERK Leptin-p38MAPK-Akt-COX-2 Leptin-caveolin-1-ERK-eNOS Leptin-Akt-eNOS Leptin-NF-κB	(75, 77–82)
Chemerin	↑proliferation ↑monocyte adhesion ↓apoptosis ↑oxidative stress ↓eNOS ↑angiogenesis ↑inflammation ↓inflammation	Chemerin-Akt/eNOS-NO Chemerin-PI3K/Akt Chemerin-ROS Chemerin-MAPK(MEK-ERK1/2,p38MAPK) Chemerin-NF-κB	(83–90)
Resistin	↑inflammation ↑monocyte adhesion ↑oxidative stress ↓eNOS ↑angiogenesis	Resistin-VLA-4-VCAM-1 Resistin-MAPK(p38/JNK/ERK1/2) Resistin-NF-κB Resistin-SOCS3-STAT3-FKN-CX3CR1-MCP-1	(91–98)
FABP4	↑inflammation ↑oxidative stress ↓angiogenesis and endothelial cell migration ↓eNOS activity and NO production ↑monocyte adhesion	FABP4-SDF-1 FABP4-eNOS FABP4-IRS1-Akt-eNOS FABP4-ERK-JNK-STAT1 FABP4-SCF/c-kit	(99–103)
RBP4	↑apoptosis ↓NO production ↑inflammation ↑oxidative stress	RBP4-P13K-AKT RBP4-ERK1/2 RBP4-TLR4-NF-κB/NADPH	(104–107)
LCN2	↑eNOS uncoupling ↑oxidative stress ↑inflammation ↑monocyte adhesion	LCN2-PKB-eNOS LCN2-NF-κB	(108–112)
Adipsin	permeability ↑inflammation ↑activation		(113, 114)

↑, increase; ↓, decrease

### 3.2 Chemerin

The two main receptors for the adipokine chemerin, which is extensively expressed in adipose tissue, are chemR23 and CCRL2 (83). In patients with essential hypertension, elevated circulating chemerin levels have been linked to early atherosclerotic alterations and endothelial dysfunction. And elevated levels of chemerin have been a reliable indicator of both heightened arterial stiffness and compromised endothelial function (115).

Chemerin induces endothelium-dependent vasorelaxation impairment via NO/cGMP signaling. Chemerin reduces vasorelaxation by decreasing NO production, enhancing NO catabolism, and decreasing NO-dependent cGMP signaling through eNOS uncoupling, and decreased GC activity (84).

Chemerin produced more ROS and phosphorylated MAPK, which enhanced the expression of pro-inflammatory mediators and monocyte adhesion to endothelial cells. In human microvascular endothelial cells, chemotherapy decreased eNOS activity and NO production. Chemerin exerts pro-apoptotic, pro-inflammatory, and pro-proliferative effects in human vascular cells through activation of Nox and redox-sensitive MAPK signaling (85). Neves et al. discovered that chemerin decreases eNOS phosphorylation and NO generation in insulin-induced endothelial cells, as well as insulin-induced vasodilation in diabetic obese mice via a mechanism of chemR23 activation, disruption of PI3K/Akt signaling, and oxidative stress (86). Chemerin/chemR23 induces increased mitochondrial ROS production in HAEC, which then triggers angiogenesis and autophagy via the AMPKα and beclin-1 pathways (87).

Chemerin promotes angiogenesis through the MAPKs pathway. Kaur et al. found that chemerin dose-dependently activated PI3K/Akt and MAPKs (p38MAPK/ERK1/2) pathway to induce the proliferation, migration, and capillary formation of ECs (88). Angiogenic effects of chemerin depending on MEK1 activity and chemerin activates ERK1 and ERK2 upon binding to CMKLR1, a receptor of the MAPK pathway (p42/44) (89). Meanwhile, Lobato et al. found that chemerin increased PE- and ET-1-induced vasoconstrictor responses through MEK-ERK1/2 pathway activation (116).

During atherogenesis, Chemerin is recruited to facilitate leukocyte adherence, when disrupted blood flow upregulates CCRL2 expression in localized vascular areas. Chemerin also activates β2 integrins in monocytes and downstream ERK1/2 through its PDI-like action (83). ChemR23 expression levels in vascular endothelial cells are regulated by inflammatory cytokines such as TNF-α, IL-1β (87), while ChemR23 upregulates these pro-inflammatory cytokines in human ECs (88). Under low-grade inflammation, IL-6 may affect endothelial eNOS expression leading to reduced NO bioavailability and producing endothelial dysfunction (117).

However, there are contradictions regarding the inflammation-inducing effects of chemerin. Yamawaki et al. found that chemerin inhibits NF-κB and p38 signaling by activating the Akt/eNOS/NO pathway. It also reduces TNF-α-induced VCAM-1 expression and promotes monocyte adherence, all of which have an anti-inflammatory impact (90). Kengo Sato et al. found that chemerin-9, an analog of chemerin, significantly reduced TNF-α-induced HUVEC adhesion and pro-inflammatory molecule mRNA expression, as well as THP1 monocyte adherence to the inflammatory phenotype of HUVEC and macrophages (118) (**Table 2**, Chemerin).

### 3.3 Resistin

According to recent research, resistin plays a variety of roles in lipid buildup, inflammation, and endothelial damage. Serum resistin levels are higher in individuals with premature coronary artery disease (PCAD) than in the general population (91). Higher resistin levels are linked to a higher risk of major adverse cardiac events (MACE) in individuals with peripheral vascular disease (PAD). In patients with PAD, resistin may serve as a marker or effector of compromised vascular physiology and unfavorable cardiac outcomes (119). In HUVECs, exogenous resistin significantly raised the levels of ET-1 and PAI-1, affecting endothelial function and VSMC migration (120).

Resistin promotes atherosclerosis progression by increasing monocyte-endothelial cell adhesion through upregulation of α4β1 integrin (VLA-4) and VCAM-1 (92). In comparison to WT mice, the aorta of ApoE-/-mice had higher amounts of resistin, resistin induced increased expression of MCP-1 and sVCAM-1 (91). Additionally, resistin elevated the production of VCAM-1 and inflammatory cytokines, but not MCP-1, in the carotid arteries of the atherosclerosis model rabbit (93). Hsu et al. found that resistin increased the expression of ICAM-1 and VCAM-1 through the p38MAPK pathway, and monocyte adhesion to HUVEC (121). Resistin increases the expression of SOCS3 in HEC and triggers the transcription factor STAT3. Inhibiting SOCS3 function stops resistin from inducing the expression of P-selectin and fractalkine, two cell adhesion molecules that limit endothelial cell activation (94). Human atherosclerotic lesions have elevated CCL19 levels. Toll-like receptor 4 (TLR4) stimulation by resistin triggers CCL19 production and NF-κB activity in HAEC (95).

Resistin induces oxidative stress, which promotes vascular lesion formation by interacting with TLR4, inducing activation of p38MAPK and NADPH oxidases, increasing the permeability of HCAEC monolayers, and increasing ROS production (96). On the other hand, resistin directly suppresses eNOS in HCAEC by inducing oxidative stress and activating JNK and p38 (97). Vascular disorders associated with angiogenesis may be significantly influenced by resistin. Human endothelial cells are stimulated to proliferate and migrate by resistin, which also encourages the development of capillary-like tubes, increases the production of MMPs and VEGFRs, and activates the ERK1/2 and p38 pathways (98) (**Table 2**, Resistin).

### 3.4 FABP4

The cytoplasmic fatty acid chaperone FABP4, often referred to as aP2, is mostly expressed in myeloid and adipocyte cells.

FABP4 functions as an intracellular fatty acid chaperone in fatty acid transport, metabolism, and signaling. It has been found to be involved in endothelial dysfunction via many routes, such as the ERK/JNK/STAT-1, eNOS, and SDF-1 signaling pathways. In endothelial cells, FABP4 interacts with CK1, influencing the transport of fatty acids mediated by eFABP4 and subsequently activation of inflammatory and oxidative stress processes (99). Ectopic expression of FABP4 has been linked to arterial neointimal formation following intravascular injury. Notably, neointima formation Interestingly, neointima development was considerably lower in FABP4-/-mice than in WT mice. Furthermore, HUVEC lacking in FABP4 showed reduced motility, a higher propensity for death, and compromised development of capillary networks. Ectopic production of FABP4 via the VEGF/VEGFR2 pathway enhances the pathogenesis of atherosclerosis and vascular damage under pathological conditions (ischemia, hypoxia) (122). VEGFA induces FABP4 in endothelial cells. Arjes et al. found that VEGFA induces FABP4 by a DLL4-NOTCH-dependent mechanism and may be regulated by the AKT pathway through FoxO1 (123).

In contrast, when FABP4 acts as an adipokine, it participates in the inflammatory response by promoting adhesion of endothelial cells to monocytes extracellularly through upregulation of adhesion molecules. FABP4 overexpression significantly increased the levels of inflammatory cytokines and adhesion molecules, preventing HCAECs from phosphorylating NOS3 (100, 101). FABP4 activation prompts the adhesion of HCAECs to monocytes by upregulating ICAM-1, VCAM-1, and P-selectin. This action inhibits eNOS phosphorylation and diminishes SDF-1 protein expression, thereby impeding vascular formation and migration (102). Moreover, FABP4’s propathogenic and angiogenic actions in the EC are largely mediated by the SCF/c-kit pathway (101). FABP4 causes insulin-mediated changes in the eNOS pathway by blocking IRS1 and Akt activation. These changes significantly reduce eNOS expression, activation, and NO generation, which eventually results in endothelial dysfunction (103) (**Table 2**, FABP4).

### 3.5 RBP4

Hepatocytes and adipocytes release retinol-binding protein 4, or RBP4, a developing adipokine. It acts as the main bloodstream carrier of vitamin A, or retinol (104). It was discovered that there was an inverse relationship between carotid intima-media and plaque echogenicity, suggesting a potential link between RBP4 and the onset of atherosclerosis (124).

RBP4 induces oxidative stress in endothelial cells, triggering an inflammatory response. RBP4 overexpression in mice and exogenous RBP4 in HAEC both cause the production of mitochondrial superoxide, which compromises the integrity and content of the mitochondria as well as the membrane potential and ultimately results in endothelial mitochondrial dysfunction. This dysfunction is accompanied by apoptosis, linked to impaired PI3K/AKT signaling pathway (105). RBP4 stimulates HUVEC to produce proinflammatory chemicals, which in turn helps humans’ leukocyte adhesion and recruitment to the endothelium through NF-κB and NADPH oxidase-dependent processes. These molecules, including VCAM-1, ICAM-1, E-selectin MCP-1, and IL-6, contribute to inflammation in HUVECs (104) RBP4.

### 3.6 LCN2

Neutrophophil gelatinase-associated lipocalin (NGAL), or lipocalin 2 (LCN2), is a glycoprotein that was recently shown to be a cytokine generated from adipose tissue. Compared to nonatherosclerotic carotid arteries, atherosclerotic carotid arteries had higher levels of LCN2 expression (108, 109).

Studies have demonstrated that LCN2 induces endothelial dysfunction by promoting eNOS uncoupling and COX production (110). LCN-2 deficiency prevents the eNOS dimer-monomer transition in the aorta, enhances activation of the PKB/eNOS pathway, and improves insulin sensitivity. On the other hand, the presence of LCN-2 exacerbates endothelial oxidative stress by facilitating ACh-induced ROS production (110). Song et al. elucidated that LCN2 buildup in arteries causes endothelial dysfunction by upsetting the vascular system and changing the polyamine balance. It may also have an effect on the eNOS-NO metabolic pathway (111).

Treatment with recombinant human LCN2 (rhLCN2) significantly increases pro-inflammatory cytokines in HUVEC, fostering an inflammatory milieu conducive to atherosclerotic lesions (109). Meanwhile, LCN2 upregulates ICAM-1, VCAM-1, and E-selectin associated with the upregulation of NF-κB in HUVEC, which enhanced THP1 monocyte adhesion to HUVEC (112). Early in the development of a lesion, a deficit in LCN2 causes an increase in plaque size, which could be the result of an increase in inflammatory monocytes. However, in advanced lesions, LCN2 deficiency reduces the formation of necrotic cores in advanced lesions. In mice with atherosclerosis, LCN2 absence lowers MMP-9 activity, which may encourage the development of more stable atherosclerotic plaques (108). Furthermore, compared to normal individuals, patients with obesity have higher circulating levels of LCN2, and variations in LCN2 are inversely linked with variations in vasodilator function. Consequently, LCN2 holds promise as a potential biomarker for obesity risk stratification (125) (**Table 2**, LCN2).

### 3.7 Adipsin

Adipsin has a role in the activation of the complement replacement pathway and is mostly secreted by adipocytes, monocytes, and macrophages. The function of adipsin in endothelial injury and atherosclerosis is now under debate.

Zhang et al. found that adipsin stimulates cell migration in CMEC angiogenesis. Adipsin can bind to Csk, thereby inhibiting the phosphorylation of Src and VE-cadherin, thus preventing the internalization of VE-cadherin. Adipsin maintains the integrity of the endothelial barrier by inhibiting VE-cadherin internalization, thereby attenuating diabetic injury-induced CMEC hyperpermeability (113).

However, it has also been shown that adipsin adversely affects cardiovascular function. Higher plasma adipsin concentrations in cardiovascular disease may trigger complement activation. Complement activation can then be mediated through receptors on immune cells and endothelial cells for allergenic toxins C3a and C5a, which cause inflammation and endothelial dysfunction. The complement system is activated when inflammatory cytokines are expressed more often and immune cells are attracted to endothelial cells (ECs) by chemotaxis. This process further suppresses endothelial NO production and causes or worsens vascular dysfunction (114, 126). However, whole body adipsin KO mice didn’t affect the development of atherosclerosis (127) (**Table 2**, Adipsin).

## 4 Discussion

**Figure 2:**
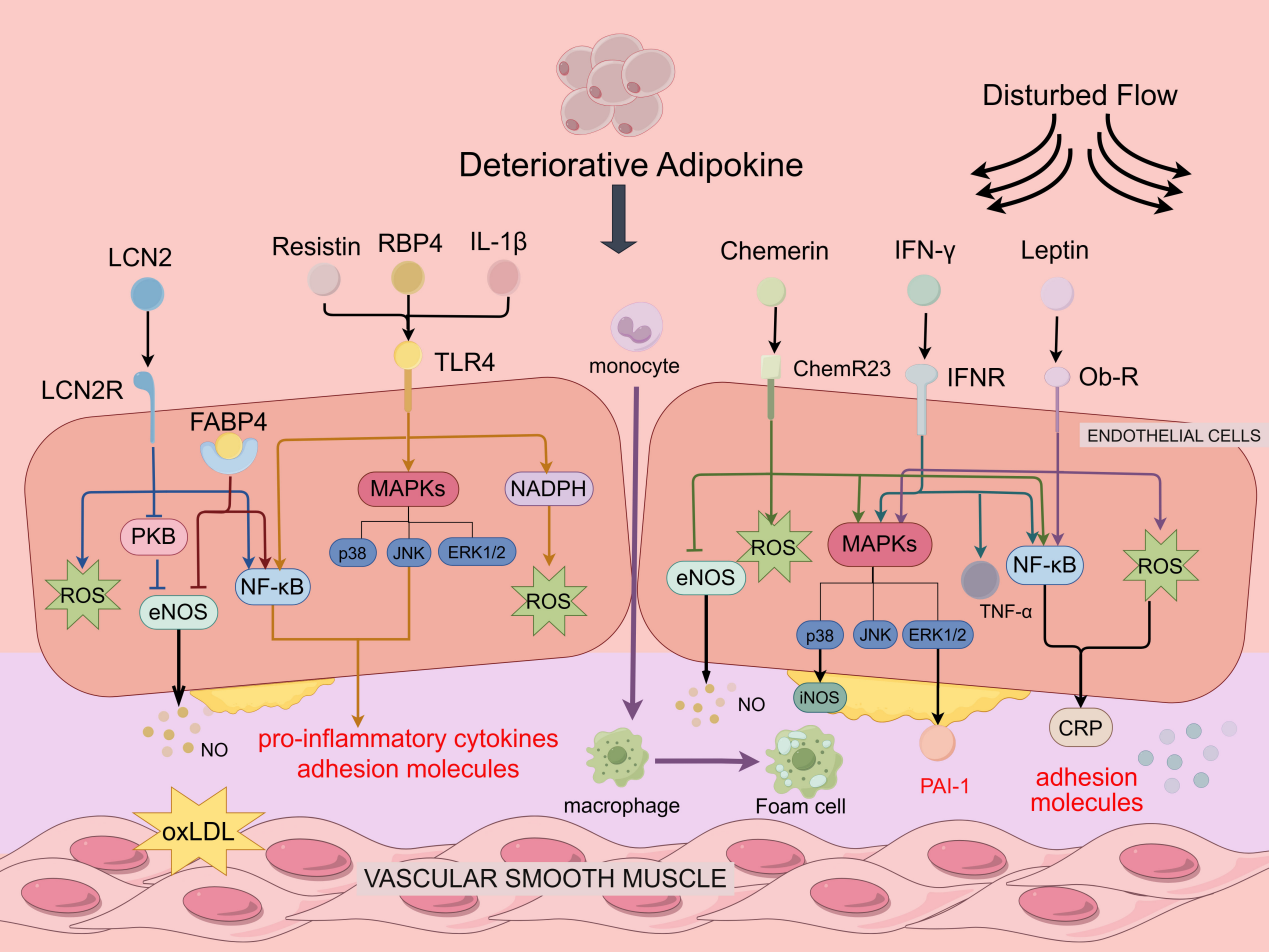
Deteriorative adipokines in regulating endothelial function and atherosclerosis. Deteriorative adipokines depicted are Leptin, Chemerin, Resistin, LCN2, RBP4, IL-1β and IFN-γ. Resistin, RBP4, IL-1β. Leptin, Chemerin, Resistin, LCN2, RBP4, IL-1β, and IFN-γ all activate the NF-κB pathway, resulting in the production of pro-inflammatory cytokines and adhesion molecules. Leptin, Chemerin and IFN-β bind to the receptor to increase ROS production, upregulate PAI-1 expression in vascular endothelial cells through the ERK1/2 pathway to promote plaque formation, activate p38MAPK to accelerate EC proliferation and capillary formation. Furthermore, IFN-γ enhanced leukocyte adhesion and recruitment by upregulating TNF-α expression in vascular endothelial cells. Resistin, RBP4, IL-1β can activate the MAPKs pathway and induce the activation of NADPH oxidase to promote ROS production by binding to TLR4. FABP4 increases inhibition of eNOS phosphorylation to suppress NO production, LCN2 inhibits NO production through the PKB/eNOS pathway and generates ROS to promote oxidative stress.

Given the rising incidence of obesity-related cardiovascular diseases, there has been growing recognition of the significance of adipokines in the progression of atherosclerosis (14). Adipokines exert a notable influence on endothelial cell regulation throughout the course of atherosclerosis development. Although various adipokines employ distinct regulatory mechanisms on endothelial cells, they also share common pathways (**Figure 1**). Protective adipokines such as Adiponectin, CTRP9, and FGF21 exert their effects by activating AMPK, phosphorylating eNOS and promoting NO production (128–130). Furthermore, these adipokines, along with PGRN, inhibit the NF-κB pathway by binding to its receptor, thereby mitigating the inflammatory response (131). Conversely, deteriorative adipokines activate the NF-κB pathway to promote the expression of adhesion and pro-inflammatory molecules (**Figure 2**). Leptin, IL-1β, Resistin, Chemerin, and LCN2 all induce oxidative stress by generating ROS (84, 85, 110, 132, 133). However, the molecular mechanisms behind the role of adipokines in regulating endothelial function in atherosclerosis have not been fully elucidated, and more research needs to be focused on this in the future.

The regulation of endothelial function by certain adipokines remains contentious. For instance, Leptin demonstrates an acute endothelium-dependent vasodilatory effect but can contribute to endothelial deterioration over time (79, 82). Similarly, RBP4 initially exerts an acute vasodilatory effect, but prolonged exposure leads to oxidative stress and inflammation in endothelial cells (106, 134, 135). Population-based experiments investigating RBP4’s influence on atherosclerosis yield conflicting results. Regarding Chemerin, its impact on endothelial cell inflammation remains uncertain. While most studies suggest proinflammatory effects, some researchers propose that Chemerin may exhibit anti-inflammatory properties, enhancing plaque conditions by promoting nitric oxide (NO) production in vascular endothelium (85, 90). Omentin, despite existing studies showing it to be protective of the endothelium, its role in the development of atherosclerosis is unclear. In endothelial cells, IL-18 is known to regulate leukocyte recruitment via specific adhesion molecules. While some studies explore its role in atherosclerosis development in smooth muscle cells, its direct impact on endothelial cells warrants further investigation.

In conclusion, adipocyte-secreted adipokines are crucial to the progression of atherosclerosis. Moving forward, additional research efforts are imperative to gain a deeper understanding of how obesity-related adipokines regulate endothelial cells in the context of atherosclerosis. Targeting certain adipokines may provide novel clinical applications in treating atherosclerosis related CADs.
